# No Correlation Between Interleukin-10 Gene Promoter Polymorphisms and Hepatitis B Virus Infection Outcome

**DOI:** 10.5812/hepatmon.8803

**Published:** 2013-05-19

**Authors:** Masoomeh Sofian, Ebrahim Kalantar, Arezoo Aghakhani, Soudabeh Hosseini, Mohammad Banifazl, Ali Eslamifar, Ali jourabchi, Ali Asghar Farazi, Amitis Ramezani

**Affiliations:** 1 Tuberculosis and Pediatric Infectious Research Center, Arak University of Medical Sciences, Arak, IR Iran; 2 Gholhak Laboratory, Tehran, IR Iran; 3 Department of Clinical Research, Pasteur Institute of Iran, Tehran, IR Iran; 4 Iranian Society for Support Patients With Infectious Disease, Tehran, IR Iran; 5 Department of Infectious Disease, Arak University of Medical Sciences, Arak, IR Iran

**Keywords:** Hepatitis B virus, Genotype, Interleukin-10, Haplotypes

## Abstract

**Background:**

Single nucleotide polymorphisms (SNP) in the promoter region of the interleukin (IL)-10 genes have a role in determining hepatitis B virus (HBV) outcome.

**Objectives:**

This study evaluates the correlation between HBV infection and SNP in IL-10 gene promoter.

**Patients and Methods:**

Ninety-six HBV-infected patients (32 chronic hepatitis B infection patients, 34 healthy carriers, 30 spontaneously recovered cases) and 31 healthy controls were enrolled. Three biallelic (-819,-592,-1082) regions in the IL-10 gene promoter were sequenced for all patients.

**Results:**

Genotypes and haplotypes of IL-10 gene promoter region at position -1082, -819 and -592 were not significantly different among controls, HBV recovered cases, carriers and chronic HBV patients. Nevertheless, A/A genotype at position -592 and T/T genotype at position -819 were more frequently seen in the HBV clearance group, while frequency of G/G genotype at position -1082 was more prevalent in the persistence group. GCC/GCC and GCC/ACC haplotypes were significantly observed in anti-HBe positive individuals.

**Conclusions:**

Our findings showed that IL-10 promoter polymorphisms were not correlated with HBV infection prognosis. Nevertheless, individuals carrying high and intermediate producer of IL-10 haplotypes had a better ability to develop anti-HBe than low producer carriers.

## 1. Background

The mechanisms of susceptibility to chronic hepatitis B virus (HBV) infection are not well clarified (1-3). Host genetic and viral factors are strong components in determining the outcomes of HBV infection (1). There is some evidence that the capacity for cytokine production in individuals may predict the progression of HBV infection (4-6). Interleukin (IL)-10 is an important anti-inflammatory cytokine secreted by several cells including T regulatory lymphocytes, activated macrophages and T helper (Th) 2 cells. The levels of IL-10 production determine immune regulation and the balance between the cellular and humoral responses (7). Several polymorphisms within the IL-10 gene promoter region have been described, including positions -1082, -819, and -592 (8). The -1082 G/G genotype is associated with high IL-10 production, but the -1082 G/A and A/A are correlated with intermediate and low production, respectively. Polymorphisms at position -819 and -592 have no independent influence on IL-10 production (9). The T and C alleles at position -819 of IL-10 promoter gene were totally in linkage disequilibrium with the IL-10-592 A and C alleles, respectively. The A allele at position-592 was solely relevant to the -1082 A allele. These make three different haplotypes: GCC, ACC and ATA (10).

## 2. Objectives

There are contradictory reports about the exact effects of IL-10 promoter gene polymorphisms on the natural outcome of HBV infection (11-14). The present study was undertaken in a central Iranian city and aimed to evaluate the correlation between HBV infection and single nucleotide polymorphisms (SNP) in the promoter regions of the IL-10 gene.

## 3. Patients and Methods

### 3.1. Study Population

96 HBV infected patients (58 males, 38 females) who were admitted to a hepatitis referral center in a city located in central Iran (Arak), and 31 matched healthy controls from a blood transfusion organization were enrolled consecutively in this study, from May 2011 to July 2011. They included 32 chronic hepatitis B patients (CHB), 34 healthy carriers, and 30 recovered cases. HBV recovery was defined as patients with negative hepatitis B surface antigen (HBsAg), positive hepatitis B core antibody (anti-HBc), and positive hepatitis B surface antibody (anti-HBs). The healthy carrier group was defined as individuals with a positive HBsAg for more than six months. The chronic hepatitis B (CHB) group was defined as a positive HBsAg for more than six months and elevated aminotransferases [ ≥ 2 times the upper limit of normal (< 35IU/L)]. The healthy carriers and CHB groups were merged together and considered as the persistent group in this study. All of the cases were negative for human immunodeficiency virus (HIV), hepatitis C virus (HCV) and hepatitis D virus (HDV) as measured by ELISA. This project was approved by the Arak University of Medical Sciences ethics committee and signed informed consent was gathered from all patients before study initiation.

### 3.2. Serological Testing

Hepatitis B surface antigen (HBsAg), antibodies to HBsAg (anti-HBs) and hepatitis B core antigen (anti-HBc) were tested by enzyme-linked immunosorbent assay (ELISA) using a commercial Kit (Hepanostika, bioMerieux, Boxtel, Netherlands). All Samples were also checked for hepatitis B e antibody (anti-HBe), hepatitis B e antigen (HBeAg) and hepatitis C antibody (anti-HCV) using ELISA. The commercial kits used were as follows: anti-HCV (Bio-Rad Laboratories, Segrate, Italy) and HBeAg and anti-HBe (Radim, Barcelona, Spain). Anti-HCV reactivity was confirmed by recombinant immunoblot assay (RIBA Innogenetics, Ghent, Belgium). All samples were tested for anti-HDV antibodies by the ELISA kit (Dia.Pro Diagnostic Bioprobessri, Milano, Italy). HIV-antibody status was assessed by ELISA (MP Biomedicals, Illkirch, France) and positive tests were confirmed by the Western blot assay (Diaplus, San Francisco, USA).

### 3.3. IL-10 Genotyping

Genomic DNA was extracted from 300μl of whole blood from each patient using the QIAamp DNA Mini Kit (Qiagen, Hilden, Germany) according to the manufacturer’s instructions. The three biallelic IL-10 promoter polymorphisms were detected by PCR using primers that amplified a short fragment of DNA containing the polymorphisms sites in combination with direct sequencing. The sequence of primers used in the PCR provided by Metabion international AG (Martinsried, Germany) was as follows:

IL10 −1082 F: 5′-ACACACACACAAATCCAAG-3′

R: 5′-ATAGGAGGTCCCTTACTTTCCTC-3′

IL10 –819 F: 5′-GAAACCAAATTCTCAGTTGGC-3′

R: 5′-ATGACCCCTACCGTCTCTATTT-3′

IL10 –592 F: 5′-AAATCGGGGTAAAGGAGC-3′

R: 5′-AGCAGCCCTTCCATTTTACT-3′

We then electrophoresed the PCR products and visualized them on a gel documentation system. The final PCR products were purified [High Pure PCR product purification kit (Roche Diagnostics GmbH, Mannheim, Germany)] and sequenced by the ABI sequencer.

### 3.4. Statistical Analysis

Statistical tests were used including the Chi-square and Fisher’s exact test along with the SPSS 16.0 data analysis software package; P < 0.05 were considered as statistically significant. The odds ratio (OR) and the 95% confidence interval (95 % CI) were calculated. Logistic regression analysis was also used to determine the correlation of a specific genotype and/or haplotype with HBV infection outcome.

## 4. Results

### 4.1. IL-10 Promoter Polymorphisms in HBV Infected Patients and Healthy Controls

IL-10 gene promoter genotype and haplotype at position -1082, -819 and -592 were checked for the HBV infected patients, recovered HBV cases and healthy controls. There was no significant difference between these groups regarding IL-10 gene promoter genotype and haplotype distribution (Table 1).

**Table 1.** Association Between IL-10 Gene Promoter Polymorphism in Different Study Groups

Genotype	Chronic HBV Infected Group, No. (%) (n = 32)	HBV Carrier Group, No. (%) (n = 34)	Recovered Group, No. (%) (n = 30)	Healthy Control Group, No. (%) (n = 31)	P value
**Locus, -592**					
A/A	2 (6.2)	5 (14.7)	6 (20)	4 (12.9)	NS^a^
C/A	15 (46.9)	14(41.2)	13 (43.3)	11 (35.5)	NS
C/C	15 (46.9)	5 (44.1)	11 (36.7)	16 (51.6)	NS
**Locus, -819**					
C/C	15 (46.9)	15 (44.1)	11 (36.7)	16 (51.6)	NS
C/T	15 (46.9)	14 (41.2)	13 (43.3)	11 (35.5)	NS
T/T	2 (6.2)	5 (14.7)	6 (20)	4(12.9)	NS
**Locus, -1082**					
A/A	14 (43.8)	18 (52.9)	17 (56.7)	13 (41.9)	NS
G/A	15 (46.9)	12 (35.3)	12 (40)	15 (48.4)	NS
G/G	3 (9.4)	4 (11.8)	1 (3.3)	3 (9.7)	NS

^a^Abbreviations: NS, not significant

### 4.2. Comparison of IL-10 Gene Promoter Polymorphisms Between Persistent and Recovered Groups

IL-10-1082 G/G and G/A frequency were slightly higher in the persistent HBV infection group while the -1082 A/A was more frequent in the recovered group. However the IL-10-1082 polymorphisms did not differ significantly between persistent and recovered groups (Table 2). The frequency of C/C genotype at position -592 of IL-10 gene promoter was slightly different in the persistence group than that in the HBV clearance group (45.5% vs. 36.7%); but A/A genotype at position -592 of IL-10 gene promoter was slightly higher in cases with clearance of HBV infection. 64.4% of the persistence group had C alleles in comparison with 58.3% of the recovered group. A alleles were seen in 32.6% of patients with persistent HBV infection but the frequency of this allele was 41.7% in recovered cases.

**Table 2.** Association Between IL-10 Gene Promoter Polymorphism and HBV Persistence

Genotype	Persistent Infection Group, No. (%) (n = 66)	Recovered Group, No. (%) (n = 30)	P value^a^	OR^b^	95% CI (OR)
**Locus, -592**					
A/A	7 (10.6)	6 (20)	0.219	0.475	0.144-1.559
C/A	29 (43.9)	13 (43.3)	0.956	1.025	0.429-2.448
C/C	30 (45.5)	11 (36.7)	0.421	1.439	0.593-3.493
**Locus, -819**					
C/C	30 (45.5)	11 (36.7)	0.421	1.439	0.593-3.493
C/T	29 (43.9)	13 (43.3)	0.956	1.025	0.429-2.448
T/T	7 (10.6)	6 (20)	0.219	0.475	0.144-1.559
**Locus, -1082**					
A/A	32 (48.5)	17 (56.7)	0.458	0.720	0.302-1.716
G/A	27 (40.9)	12 (40)	0.933	1.038	0.431-2.504
G/G	7 (10.6)	1 (3.3)	0.258	3.441	0.404-29.3

^a^P value < 0.05 was statistically significant

^b^Abbreviations: OR, odds ratio; 95% CI, 95% confidence interval

We found slightly different frequency of IL-10-819 T/T in the recovered group and -819 C/C in the persistent HBV infection group. However, these differences were not significant between the subjects with persistent and recovered HBV infection (Table 2). In the haplotype analysis, GCC/GCC homozygous type was found more frequently in patients with persistent HBV infection, while ATA/ATA homozygous type was more frequent in HBV clearance group. However, these differences were not significant between these groups (Table 3).

**Table 3.** Haplotype Analysis of IL-10 Gene Promoter in Persistent and Recovered Groups

Haplotype	Persistent Infection Group, No. (%) (n = 66)	Recovered Group, No. (%) (n = 30)	P value^a^	OR^b^	95% CI^b^ (OR)
**Homozygous Type**					
ACC/ACC	8 (12.1)	3 (10)	1	1.241	0.305-5.051
GCC/GCC	7 (10.6)	1 (3.3)	0.428	3.441	0.404-29.3
ATA/ATA	7 (10.6)	6 (20)	0.218	0.475	0.144-1.559
**Heterozygous Type**					
GCC/ACC	15 (22.7)	7 (23.3)	1	0.966	0.347-2.689
GCC/ATA	12 (18.2)	5 (16.7)	1	1.111	0.353-3.495
ACC/ATA	17 (25.8)	8 (26.7)	1	0.954	0.358-2.540

^a^P value < 0.05 was statistically significant

^b^Abbreviations: OR, odds ratio; 95% CI, 95% confidence interval

### 4.3. Correlation between HBeAg Seroconversion and IL-10 Genotype and Haplotype 

No significant difference was observed between the HBeAg and anti-HBe positive groups in the genotyping analysis (Table 4). Nevertheless, in the haplotype analysis, the GCC/GCC homozygous and GCC/ACC heterozygous types were more frequently found in the anti-HBe positive group (OR = 1.5, P < 0.05 and OR = 1.64, P < 0.05 respectively) (Table 5).

**Table 4.** Genotype Analysis inHBeAg Positive and Negative HBV Infected Patients

Genotype	HBeAg^a^ Positive, No. (%)	HBeAg Negative, No. (%)	P value
**Locus, -592**			
A/A	1 (14.3)	6 (10.9)	NS^a^
C/A	4 (57.1)	23 (41.8)	NS
C/C	2 (28.6)	26 (47.3)	NS
**Locus, -819**			
C/C	2 (28.6)	26 (47.3)	NS
C/T	4 (57.1)	23 (41.8)	NS
T/T	1 (14.3)	6 (10.9)	NS
**Locus, -1082**			
A/A	4 (57.1)	27 (49.01)	NS
G/A	3 (42.9)	22 (40)	NS
G/G	0 (0)	6 (10.9)	NS

^a^Abbreviations: HBeAg, hepatitis B e antigen; NS, not significant

**Table 5.** Haplotype Analysisof Anti-HBe Positive and Negative HBV Infected Patients

Haplotypes	Anti-HBe^a^ positive	Anti-HBe negative	OR^a^	95% CI^a^ (OR)
**ACC/ACC**	15%	33.3%	0.353	0.055-2.247
**GCC/GCC**	10%	0%	1.5	1.149-1.959
**ATA/ATA**	10%	11.1%	0.889	0.070-11.280
**GCC/ACC**	30%	0%	1.643	1.184-2.280
**GCC/ATA**	20%	44.4%	0.312	0.056-1.730
**ACC/ATA**	15%	11.2%	1.412	0.126-15.784

^a^Abbreviations: Anti-HBe, hepatitis B e antibody; OR, odds ratio; 95% CI, 95% confidence interval

## 5. Discussion

In the present study we examined the polymorphisms of promoter regions of IL-10 gene and the correlation of these polymorphisms with HBV clearance and disease progression. The frequency of A/A genotype at position -592 and T/T genotype at position -819 of IL-10 gene promoter were higher in the HBV clearance group than that in the persistence group. The G/G genotype at position -1082 was more prevalent in the persistence group. Individuals carrying high and intermediate producer haplotypes of IL-10 had more capacity to develop anti-HBe than low producer haplotypes. Cytokines play a critical role in modulating inflammatory and immune responses. Polymorphisms in the regulatory regions of the cytokine genes have important impact on their expression. Thus the cytokine genes polymorphisms have a major contribution in predicting disease susceptibility and clinical outcome (3). IL-10 is a crucial cytokine with immune-regulatory and anti-inflammatory effects and is associated with many diseases (15). IL-10 is produced by Th2 cells, T regulatory lymphocytes and activated macrophages and inhibits expression of other pro-inflammatory cytokines such as IFN-gamma, IL-2 and TNF-alpha in Th1 cells (16). IL-10 gene polymorphisms can affect the transcription, translation and secretion of IL-10 (15). Polymorphisms in the IL-10 promoter region and associations with HBV infection and progression have been examined widely in different populations throughout the world (7, 11-14, 17). There are three classic haplotypes in the IL-10 promoter region including GCC, ACC, and ATA (9). The GCC haplotype produces high,, ACC medium, and ATA low levels of IL-10 (18, 19). The three proximal promoter SNPs and haplotypes have been associated with hepatitis B infection outcomes in Korean (11, 12), Chinese (20-22), Japanese (23) and African American populations (9, 24). These reports suggest the important role of IL-10 variation in HBV infection outcome. Cheong et al. (11) reported that high producer genotypes of IL-10 (IL-10-592 C/C) carriers had a better capacity to recover from HBV spontaneously versus individuals with low IL-10 producer allele (IL-10 -592A). In contrast, Shin et al. (12) indicated that patients who have IL-10 ht2 (high IL-10 producers) show an accelerated course of chronic HBV infection. Turner et al (9) also reported that people with the IL-10-592 A/A genotype had lower IL-10 levels and a favorable HBV disease outcome. Miyazoe et al (5) has also found that carriers of the ATA haplotype (low IL-10 production ability) have favorable HBV-related outcomes. In another study in Iran, IL-10-592 C/C was significantly more common in patients with occult HBV infection (25). In an investigation by Peng et al. (20), patients with IL-10 intermediate producer genotypes were more likely to produce anti-HBe than IL-10 low producer genotype carriers, so HBeAg seroconversions were more frequently seen in IL-10 intermediate producer genotypes or haplotypes. In another study by Wu et al (13) the IL-10-1082 G/G genotype polymorphism was associated with earlier HBeAg seroconversion and lower HBV viral load compared to A allele carriers. Gao et al (7) showed that IL-10-1082 AA/AG did not differ significantly between subjects with HBV infection and controls. They also reported that the IL-10-1082 A/G alleles and IL-10-592 A/C polymorphisms did not differ significantly between cases and controls. A meta analysis conducted by Lu et al (15) indicated that the G and A alleles in IL-10-1082 were not associated with HBV infection in the Asian population. The study by Zhang et al. (14) showed no significant difference at position -1082G/A, -819T/C and -592A/C of IL-10 gene promoter region among normal controls, recovered HBV individuals and chronic HBV patients. Nevertheless, it was reported that T/T genotype at position -819T/C and A/A genotype at position -592A/C were significantly more frequent in chronic hepatitis B patients than in asymptomatic HBV carriers. Our results showed no significant difference in frequencies of genotypes and haplotypes of IL-10 gene promoter region at position -1082, -819 and -592 among cases with HBV infection and controls, which is in agreement with findings of Zhang et al (14), The results of the Gao et al. (7), and Lu et al (15) studies are in contrary with Miyazoe et al (5), Shin et al (12), Turner (9) and Cheong et al (11) investigations. These conflicting results may be due to the influence of other genes on HBV progression than just a direct link between IL-10 expression and HBV outcome. Moreover they can be related to epidemiological and geographical factors and study circumstances such as the characteristics and number of the patients and HBV genotype variations. Our study also showed that patients with high and intermediate producer haplotypes of IL-10 had more ability to produce anti-HBe than low producer haplotype carriers and this is in agreement with Peng et al (20) and Wu et al (13) results. These findings are in concordance with the inhibiting effects of IL-10 on pre-inflammatory cytokines and self-limiting HBV infection in high producer genotype carriers. In conclusion, our study showed that IL-10 promoter region polymorphisms may not be correlated with HBV infection prognosis. Nevertheless, individuals carrying high and intermediate producers of IL-10 haplotypes had more ability to develop anti-HBe than low producer carriers. However, due to the limited number of patients in the current study, a conclusion cannot be reached regarding the correlation of IL-10 gene promoter polymorphisms and HBV infection outcome, so further studies should be carried out to determine this association.
